# Prevalence of Active Primitive Reflexes and Craniosacral Blocks in Apparently Healthy Children and Relationships with Neurodevelopment Disturbances

**DOI:** 10.3390/children10061014

**Published:** 2023-06-04

**Authors:** Gema León-Bravo, Irene Cantarero-Carmona, Javier Caballero-Villarraso

**Affiliations:** 1 Department of Nursing, Pharmacology and Physiotherapy, University of Córdoba,14004 Córdoba, Spain; glbravo@uco.es; 2 Department of Morphological and Sociosanitary Sciences, University of Córdoba, 14004 Córdoba, Spain; b12cacai@uco.es; 3 Department of Biochemistry and Molecular Biology & UGC Clinical Analyses, University of Córdoba, 14004 Córdoba, Spain; 4 Maimonides Institute of Biomedical Research of Córdoba (IMIBIC), 14004 Córdoba, Spain; 5 Reina Sofia University Hospital, 14004 Córdoba, Spain

**Keywords:** primitive reflexes, craniosacral dysfunction, neurodevelopment, early diagnosis, screening

## Abstract

Background: In healthy children, the frequency of the anomalous persistence of primitive reflexes (PRs) and craniosacral blocks (CBs) is unknown, as well as their impact on neurodevelopment, behaviour disorders and related consequences. We aim to know the prevalence of anomalous PRs and CBs in apparently healthy children and their relationships with behavior and neurodevelopment anomalies. Methods: Participants (*n* = 120) were evaluated via a physical examination to detect PRs and CBs and an ad hoc parent survey to collect perinatal events, and children’s behavioral assessments were conducted by teachers using the Battelle score. Results: PRs were present in 89.5%. Moro (70.8%), cervical asymmetric (78.3%) and cervical symmetric PRs (67.5%) were the most frequently observed PRs. CBs were found in 83.2%, and the most frequent CBs were dura mater (77.5%) and sphenoid bone (70%) blocks. Moro, cervical asymmetric and cervical symmetric active primitive reflexes were significantly associated with cranial blocks of dura mater, parietal zones and sphenoid bone sway. Gestational disorders or perinatal complications were associated with a higher frequency of PRs and CBs. The presence of PRs and CBs was associated with abnormal Battelle scores and neurobehavioral problems. Conclusion: The presence of PRs and CBs in children without diagnosed diseases is frequent and related to disturbances in childhood neurodevelopment.

## 1. Introduction

Primitive reflexes (PRs) are automatic, involuntary, and stereotyped movements directed from the brainstem and executed without the involvement of the cerebral cortex. On the other hand, craniosacral blocks (CBs) are alterations in the movements of the cranial bones and their coordination with the sacrum due to an imbalance of membranous tensions and restrictions, causing intracranial disorders and distal affectations. PRs are indispensable for the survival of the individual in the early stages of life. They are necessary for responding in a coordinated manner to sensory stimuli during the period of cortical immaturity until the maturation of the central nervous system (CNS) [1]. However, they must gradually be abolished as the subject’s development proceeds, allowing for voluntary and functional movement responses. This process of PR abolition is due to the synaptic plasticity of the CNS. Persistent PRs can be identified by means of a systematic neurological examination. The persistence of PR in children has been related to neurodevelopment disorders, with pathological situations such as attention deficit, hyperactivity, depression, autism and poor concentration in addition to patterns of alterations in their cognitive, motor, psychological and social development [2,3,4,5].

In addition, the craniosacral system is intimately related to numerous structures, generating bidirectional influences with nervous, musculoskeletal, vascular, lymphatic, endocrine and respiratory systems. In physiological conditions, this system seeks to balance the mobility of membranes and cranial sutures based on the coordinated movement of cranial bones and sacrum blockages in the craniosacral system, which can be evidenced via craniosacral exploration detecting intracranial membranous tensions [6,7]. The presence of CB has been observed in endocrine disorders due to the alteration of sphenobasilar synchondrosis, visceral disorders due to the involvement of the vagus nerve, migraines, delayed motor behavior, stress, anxiety, bruxism and a lack of concentration, leading to different disorders that occur as the condition progresses and worsening the physical and mental wellbeing of the child. Currently, after the age of one or one and a half years, the exploration and follow-up of active primitive reflexes (APRs) are carried out in children with some diagnosed diseases [5,8]. If neurological homeostasis is disturbed in the child after diagnosing possible CB, this could manifest in the persistence of APR and vice versa.

However, there is no information about the persistence of APR and the existence of CB in apparently healthy children. It would be interesting to know this as APR and CB could affect their daily and future lives. The observation of the different behavioral patterns of the child in social and emotional areas, as well as his or her physical and psychological capacities, could help in the early detection of neurodevelopment disturbances. Such detection is essential for the health professional in order to establish an appropriate preventive treatment to avoid long-term health problems [8,9,10].

Therefore, our aim is to study the prevalence of APR and CB in apparently healthy children, analyze potential perinatal causative factors and describe the impact on neurodevelopment and educational performance.

## 2. Materials and Methods

### 2.1. Study Design and Participants

A cross-sectional, observational, descriptive study was conducted in a school population. The total study sample included 120 apparently healthy children, according to the National Health System, without neurological disability. Consequently, none of the recruited children for this research study had any neurological disease. They all belonged to the same school in our city (Córdoba, Spain). This avoided any possible cultural or linguistic bias. In addition, unhealthy children or children with any neurological disorders, children out of school and those who did not belong to the same age range were excluded. The age of the participants of both sexes ranged from 3 to 8 years.

The application of tests and data collection was carried out within the facilities of the collaborating institution for the study, with the contribution of the entire team and with authorization of management from the school center and parents or legal representatives of the children. Before beginning the study, parents and teachers were trained in the application of the Battelle scale.

### 2.2. Ethics Approval

The study was approved by the reference Research Ethics Committee (1763-N-19). This study was registered in the ClinicalTrials.gov website using study reference NCT05002504 (accessed on 29 December 2021).

### 2.3. Measurements

#### 2.3.1. Primary Outcomes

The presence of APR and CB was evaluated via a physical examination by an experienced physiotherapist according to the traditional neurological assessment and the method of Andrzej Pilat [11] and John E. Upledger [12], respectively. The method of measuring craniosacral blocks is based on palpation and fascial manipulation techniques applied between the skull and sacrum. These techniques attempt to restore movement at the level of the individual sutures of the skull, the skull itself (as a total entity) and the skull in relation to the sacrum. Initially, the aim of CB screening was to normalize sympathetic nerve activity [13].

The primitive reflexes explored were Moro reflex, cervical asymmetric, supine labyrinthine tonic, prone labyrinthine tonic, palmar grasp, plantar grasp, lateral trunk propulsion, parachute, Galant, search, cervical symmetric, Babinski, cochlea-palpebral and acoustic. These reflexes were considered inactive (0) or active (1). On the other hand, the parameters of the craniosacral system that were explored were dura mater sway, frontal bone, parietal bones, temporal bones, temporomandibular joint and sphenoid bone. These parameters were considered normal (0) or blockage (1).

#### 2.3.2. Secondary Outcomes

The type of pregnancy was classified on a 2-point scale (0 = normal; 1 = with some complication). The time of gestation and type of delivery were each classified on a 3-point scale (0 = 9 months of gestation and natural delivery; 1 = 8 months or instrumental delivery; 2 = 7 months or cesarean delivery). The classification of with/without perinatal problems reflects the sum of the scores (<2 without perinatal problems and ≥2 with perinatal problems). Characteristics related to the time of delivery (gestational age) and type of birth are shown in Table 1.

#### 2.3.3. Tertiary Outcomes

Neurobehavioral aspects were analyzed using the “Battelle Developmental Inventory” (BDI), which evaluates five areas of development and performance (personal/social, adaptive, motor, communicative and cognitive) by using a structured exam. This information is obtained via an observation guide, both in class and at home, and by using the information provided by parents and teachers [14]. The neurodevelopmental assessment was carried out by taking into account the growth stages standardized by the WHO [15]. The results are assigned in age-adjusted percentages, and they are classified as follows: low (0–50%), medium (50–80%) and high (80–100%). Thus, “low” values indicate poor overall development in one or more of the areas evaluated; “medium” values indicate normal development according to their age; finally, “high” values indicate excessive global development in one or more of the evaluated areas. Low and high values are considered impairments in one or more of the evaluated areas [16,17].

#### 2.3.4. Quaternary Outcomes

The age and anthropometric characteristics (height, weight and body mass index) of the children were also recorded (Table 2).

### 2.4. Statistical Analysis

Qualitative variables were expressed as frequencies and percentages, and quantitative variables were expressed as the mean and standard deviation. To check the normality of distributions, Kolmogorov–Smirnov tests were performed.

Finally, the chi-square test was used to analyze differences between age ranges, genders, perinatal and neurobehavioral problems, and teacher assessment versus physical therapy exploration, as well as the association between APR and CB. For all evaluated criteria, a confidence level of 95% was established by considering a value of *p* < 0.05 as statistically significant.

## 3. Results

### 3.1. Prevalence of Active Primitive Reflexes and Craniosacral Blocks

The overall mean age was 5.49 ± 1.72 years, of which 54 (45%) were girls and 66 (55%) were boys. In 89.5% of the total sample, there was evidence of the persistence of at least one of the fourteen reflexes studied, and only 10.5% of the children were free of APR. Moro (70.8%), cervical asymmetric (78.3%) and cervical symmetrical (67.5%) reflexes were the most frequent in the child population studied. The cervical asymmetric active reflex follows the natural biological evolution conditioned from infancy and disappears or diminishes, and it is inappropriate for this reflex to be active from 3 to 8 years. Surprisingly, Moro and cervical symmetric reflexes are more frequent from 6 to 8 years of age than from 3 to 5 years. There are no significant differences in terms of gender (Table 3).

In 83.2% of the sample, at least one of the six blocks considered was found and 16.8% of the children did not present CB. The study showed a high prevalence of dura mater, sphenoid bone and parietal zones block in the referred infants, but in general, all blocks were more common with increasing age. There are no significant differences in CB appearance between girls and boys (Table 4).

### 3.2. Relationship between APRs or CBs and Battelle Score

A statistically significant increase in Moro, asymmetric, symmetric, plantar pronging, cochlea-palpebral and acoustic reflexes was observed in the low and high scores of the Battelle scale compared to the normal performance group (Table 5). On the other hand, there was a highly significant effect with respect to the dura mater and sphenoid bone blocks on low and high Battelle scores (*p* < 0.001), to a lesser extent than the parietal bone (*p* < 0.05). Additionally, the sphenoid bone block is more frequent in all studied boys, but in the case of girls, parietal zones and the dura mater are the most recurrent (Table 6).

### 3.3. Neurodevelopment Outcome in Children with Perinatal Problems

Children who have experienced complications during the prenatal period, during childbirth or in the first days of birth show a considerable risk of physical, neuropsychological, cognitive and behavioral disorders, which are revealed by the Battelle scale (*p* < 0.001) (Table 7).

**Figure 1:**
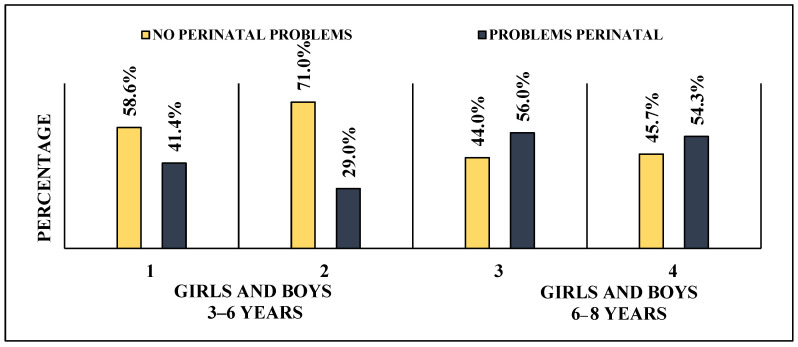
Perinatal problems in both age groups.

**Figure 2:**
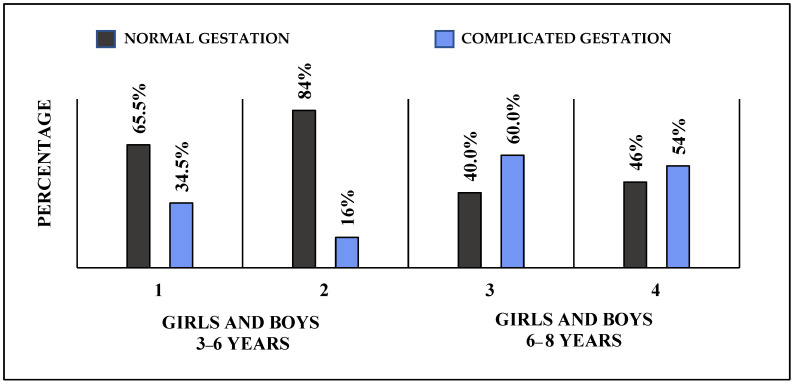
Gestational complications in both age groups.

In the 6–8 age group, 56% of girls had perinatal problems and 54.3% of boys had perinatal problems, while in the 3–6 age group, 29% of boys and 41.4% of girls had perinatal problems (Figure 1 and Figure 2).

### 3.4. Association between APRs and CBs

The physical examination of APR and CB showed statistically significant associations between symmetrical cervical, asymmetrical cervical, and Moro reflexes with the dura mater block (*p* < 0.001). These last two reflexes are also associated with the blockage of the sphenoid bone (*p* < 0.001). Likewise, there is a significant association between the asymmetric cervical reflex and the blockade of parietal bones (*p* < 0.001). Thus, when there is blockage of the dura mater, it favors the Moro and asymmetric and symmetric cervical reflexes to stay active. When a blockage of the sphenoid is detected, it causes the asymmetric cervical and Moro reflexes to stay inactive. Additionally, when the parietal bone is blocked, it keeps the asymmetrical cervical reflex active (Table 8).

## 4. Discussion

Our results show that (i) apparently healthy children can have an anomalous activation of certain PR and CB; (ii) the presence of these PRs is related to the presence of CB; (iii) the presence of PR and CB could be related to perinatal disorders; (iv) the presence of PR and CB is related to abnormal neurodevelopmental Battelle scores.

The finding of the existence of APR and CB in older children from 3 to 8 years of age (in whom such PR should be abolished and cranial blocks are non-existent, considering normal neurodevelopment) is a remarkable and interesting finding in the present study. Such reflexes showed a statistically significant association with the most frequently found CB. In view of these findings, two questions can be asked: (i) Are these PRs persistently activated from birth, or are they reactivated again or persist for some reason after being abolished? (ii) Is the relationship between APR and CB causal or consecutive?

Regarding the first question, we can state that the most common alterations in terms of APR (cervical asymmetric—the only reflex more frequent between 3 and 5 years—Moro and symmetric cervical reflexes) and CB (swaying of the dura mater and sphenoid bone) were positively associated with the age of the child, which ranges from 6 to 8 years with no sex differences. The asymmetric cervical reflex follows the natural biological evolution conditioned from infancy and disappears or diminishes, although it is still inappropriate for it to be active from 3 to 8 years of age. However, Moro and cervical symmetrical reflexes are more frequent from 6 to 8 years of age than from 3 to 5 years of age. Indeed, it is observed that this group was the one that presented more perinatal and gestational disorders at birth, which could be the reason for remaining active. These data would be of considerable interest to teachers since it would be necessary to try to apply a correction to them in their classrooms. Similarly to this study, Gieysztor et al. [17] examined trunk rotation in relation to the persistence of APR in school children of 5–9 years of age, observing trunk rotation problems associated with APR (Galant reflex). In addition, these authors conducted a second study in which they observed the occurrence of APR in healthy children aged 4–6 years and analyzed its impact on psychomotor development. Similarly, Grigg et al. [18] examined APR in children from 7 to 12 years old by means of rhythmic therapy and found that cervical asymmetric APR was associated with behavioral problems. Thus, we agree with these authors, affirming that after the age of abolition (up to 2 years old), APRs persist in children after 3 years old, altering their neurodevelopmental skills even if they are within the group of children classified as normal. However, there are no previous studies that have directly related and demonstrated APR and CB in a population of this size with perinatal variables, type and time of pregnancy, delivery, neurobehavioral factors and mental concentration in the infant. Moreover, its impact on school performance and various neurodevelopmental factors has not been studied. Risk factor assessment can be an important element in child development observation. Environmental, genetic, biological [19,20], social and demographic [21] factors may increase the risk of developmental delays; therefore, the children exposed to those factors more often require neurological assessment and observation.

It can be observed that, similarly to primitive reflexes, CBs follow the natural process of being blocked during cesarean delivery or instrumental delivery; thus, neurological affectation can occur due to perinatal problems and gestational disorders. Therefore, we detected that in the 3–5-year-old group, the cranial bones remain blocked once their fontanels have closed after birth. The sphenoid bone is the most blocked when there have been previous perinatal problems and in those born at 7 or 8 months of gestation. In cesarean deliveries, the greatest cranial involvement was observed in the dura mater and sphenoid, as these are not the natural exit route for the good mobilization of cranial bones. Additionally, in instrumental deliveries, the greatest involvement was of the dura mater because of the undue traction that could be caused by this method. In the 6–8-year-old group, there was a greater frequency of gestational and perinatal problems, which could be related to the activation of Moro and cervical symmetric reflexes. This association between gestational and perinatal disturbances with the persistence of these primitive reflexes could be a possible causal relationship. There are no previous studies that have used these variables to reach the results that we have observed. Nevertheless, the results need to be interpreted with great caution because the postnatal environment is a relevant possibility for the increased risk of these disturbances.

According to the Battelle scale, children with high and low performances that are considered abnormal are directly associated with the existence of APR and CB.

With respect to the second question, regarding the relationship between the most common APR and the most frequent CB, we have found an association between Moro, cervical asymmetric and cervical symmetric reflexes with respect to the sway blocks of the dura mater, sphenoid bone and parietal bone. Such a finding may suggest that dysfunctions in these cranial systems somehow contribute to the persistence of these reflexes. Thus, we agree in part with H. Haller et al. [22], who determined the effectiveness of craniosacral examination and subsequent therapy in individuals with chronic nonspecific cervical problems based on the detection of the dura and cranial bone sway blocks. We also agree with the results of another study by Niklasson et al. [23], who compared apparently healthy children (in terms of psycho-behavioral maturation) to children presenting developmental disorders by means of a systematic physiotherapeutic evaluation, demonstrating that despite them being children that supposedly do not have pathologies, they could have some underlying developmental disorder that was previously undiagnosed.

Perinatal problems, such as pregnancies with some degree of difficulty or children born preterm (specifically those born at 7 or 8 months), were associated with the presence of APR and CB, as were instrumental deliveries or cesarean births, which were directly associated with the presence of blockages in the sway of the dura mater and sphenoid bone. Gieysztor et al. [24] obtained similar information, observing that preterm infants show a higher level of APR associated with a lower level of psychomotor skills.

On the other hand, there is a significant direct relationship between the neurobehavioral problems of the children (obtained through the parent survey) and the APR and/or CBs. If these results are compared with the results of the BDI for neurodevelopment obtained by the teachers, we see that they are along the same lines, and it can be said that the same results are obtained by using different assessment methods. Thus, it can be stated that the persistence of asymmetric and symmetric cervical APRs together with the CB of the dura mater, parietal areas and sphenoid bone is coincident in the two relationships. As the most relevant neurobehavioral problem, we obtained results of low or very low concentration abilities (via the parents’ survey) that corresponded with low and high BDI values from the teachers’ assessment. Therefore, we agree with Alamiri et al. [25], who observed abnormal signs of psycho-neurological development in children and their relationship with low cognitive performance, suggesting that such neurobehavioral problems are possibly due to poor neurodevelopmental monitoring during early childhood. A study by Konicarova and Bob [26] showed the relationship between cervical asymmetric APR and attention-deficit hyperactivity disorder (ADHD) in schoolchildren.

One of the possible limitations of this research study is that a gender-stratified analysis has not been carried out. This would have required a larger sample size. Another limitation of cross-sectional studies is that we have compared two groups globally at a specific moment in time, but in order to obtain causal associations with greater robustness, it would be necessary to follow the evolution of each subject. Therefore, future research should be carried out on larger population samples and longitudinal studies as a comprehensive follow-up could provide more information, especially of a causal nature. Another possible limitation could be that this study was conducted in a single center. If it had been carried out in several centers and different geographical regions, the external validity of the results would have increased. In addition, this would contribute to the increase in the sample size mentioned above. However, it would be necessary to control for another possible limitation, which would be the fact that the measurement methods used (Battelle scale and CB and APR detection) cannot be automated. That is, since they cannot be carried out by means of calibrated devices, there could be some inter-observer variability. Therefore, if multicenter studies involving different observers were to be carried out, it would be advisable to perform a stratified analysis of the results and consider the inter-observer Kappa coefficient.

Based on the results obtained, we advocate the systematic evaluation of the persistence of active primitive reflexes in children from 3 to 8 years old, as well as of craniosacral tensions and blocks that may contribute to the maintenance of such reflexes. In this way, we would be able to detect early imbalances in these APRs and CBs, and we would be able to correct them in time, avoiding major long-term complications that may lead to a possible deficit in children’s neurodevelopment. Thinking about the possible future implications of this study, we believe that the obtained results could lead (at least in part) to designing screening programs for the early diagnosis of these neurodevelopmental disorders in children. These screening programs could be implemented in the future in primary care centers or schools. Based on the early detection of these problems, appropriate treatment should be given, and the results shown by the children after treatment should be observed. Future research should be focused on the follow-up of children who are detected with any of these disorders and who undergo treatment in order to check how they evolve. Currently, the authors of this research study are carrying out the treatment and follow-up of the children who were diagnosed during this study. Therefore, it should be noted that the results shown here are, to some extent, provisional, pending the completion of the follow-up study, which may or may not corroborate some of the comments provided here.

## 5. Conclusions

After analyzing the prevalence of APR and CB in the healthy population, it can be concluded that these situations are highly present in regular education schools. In addition, it can be observed after this study that the presence of APR and CB influences child neurodevelopment in general at cognitive, social, physical and emotional levels.

Consequently, establishing systematic physical examination programs is recommended, and examining the global development of a child with respect to psychomotor and intellectual facets is also recommended.

## Figures and Tables

**Table 1:** Children’s gestational and perinatal characteristics.

	Children from 3 to 5 Years		Children from 6 to 8 Years	
	$\bar{x}$	SD	$\bar{x}$	SD
**Age** (years)	4.0	0.8	7	0.82
**Height** (cm)	102.4	4.5	120	6.13
**Weight** (kg)	16.8	4.5	25.83	2.87
**BMI** ^a^ (kg/m^2^)	15.8	3.4	17.88	1.91

^a^ BMI: Body mass index.

**Table 2:** Children’s anthropometric characteristics.

	Delivery Type			Birth Week		
	Vaginal	Cesarean	Instrumental	9 Months	8 Months	7 Months
**Children** **3–5 years**	37 children	20 children	3 children	44 children	15 children	1 child
**Children** **6–8 years**	28 children	22 children	10 children	27 children	25 children	8 children

**Table 3:** Prevalence of active primitive reflexes by sex and reflex type ^a^.

Active Primitive Reflexes	Overall (*n* = 120)	3–5 Years (*n* = 60)		6–8 Years (*n* = 60)	
		Girls	Boys	Girls	Boys
Moro	70.8	18.4	17	26.5	20.3
Cervical asymmetric	78.3	23.6	26.8	27.9	14.9
Supine labyrinthine	16.7	6.1	3.6	1.5	6.1
Labyrinthine prose	16.7	4.1	5.4	7.4	3.2
Palmar pronation	7.5	1	4.5	0	2
Plantar pronging	18.3	2	6.3	4.4	6.7
Placement	10	2	2.7	1.5	4.1
Parachute	21.7	9.2	5.4	1.5	6.8
Galant	8.3	3.1	3.2	1.5	1.4
Search	2.5	0	0	1.5	1.4
Cervical symmetric	67.5	21.4	18.8	19.1	23.6
Babinski	6.7	7.1	6.3	4.3	8.1
Cochleopalpebral	24.2	1	0	2.9	0.7
Acoustic	3.3	1	0	0	0.7

^a^ Prevalence is expressed as percentage, balanced for age group within sex.

**Table 4:** Prevalence of craniosacral blocks by sex and block type ^a^.

Craniosacral Blocks	Overall (*n* = 120)	3–5 Years (*n* = 60)		6–8 Years (*n* = 60)	
		Girls	Boys	Girls	Boys
Dura mater	77.5	21.5	19.1	22.8	20.2
Frontal bone	10.8	5.9	4.8	6.3	4.9
Parietal bone zones	30.8	16	9.9	16.8	8.2
Temporal bone zones	13.3	9.9	8.9	7.8	8.9
ATM	14.2	16.8	9.8	15.3	9
Sphenoid bone	70	29.9	47.5	31	48.8

^a^ Prevalence is expressed as percentage, balanced for age group within sex.

**Table 5:** Prevalence of active primitive reflexes by Battelle score (*n* = 120) ^a^.

Active Primitive Reflexes	Battelle Score			*p*-Value
	Low Performance	Normal Performance	High Performance	
Moro	84	30.6	60.9	<0.001
Cervical asymmetric	92	26.4	65.2	<0.001
Cervical symmetrical	80	16.7	91.3	<0.001
Supine labyrinthine	16	16.7	17.4	0.99
Labyrinthine prose	20	18.1	8.7	0.51
Palmar pronation	4	9.7	4.3	0.53
Plantar pronging	20	11.1	39.1	0.01
Placement	8	6.9	21.7	0.11
Parachute	24	20.8	21.7	0.95
Galant	8	6.9	13	0.65
Search	0	2.8	4.3	0.61
Babimski	20	19.4	43.5	0.06
Cochleopalpebral	0	1.4	13	0.02
Acoustic	52	31.9	82.6	<0.001

^a^ Prevalence is expressed as percentage, balanced for age group within sex.

**Table 6:** Prevalence of active craniosacral blocks by the Battelle score (*n* = 120) ^a^.

Craniosacral Blocks	Battelle Score			*p*-Value
	Low Performance	Normal Performance	High Performance	
Dura mater	80	38.9	87	<0.001
Frontal bone	4	8.3	26.1	0.06
Parietal bone zones	72	45.8	69.6	0.04
Temporal bone zones	4	11.1	30.4	0.12
ATM	16	18.1	0	0.09
Sphenoid bone	84	33.3	95.7	<0.001

^a^ Prevalence is expressed as a percentage and balanced for age groups within sex.

**Table 7:** Prevalence of perinatal problems by the Battelle score (*n* = 120) ^a^.

Perinatal Problems	Battelle Score			*p*-Value
	Low Performance	Normal Performance	High Performance	
**Present**	95	7	85	<0.001
**Absent**	5	93	15	<0.001

^a^ Prevalence is expressed as a percentage.

**Table 8:** Frequencies and associations between active primitive reflexes and cranial blocks (*n* = 120).

		Dura Mater				*p*	Parietal Zones				*p*	Sphenoid Bone				*p*
		N		B			N		B			N		B		
APR FREQUENT		AF	RF	AF	RF		AF	RF	AF	RF		AF	RF	AF	RF	
**Moro**	Inactive	21	17.5	14	11.7	<0.001	21	17.5	14	11.7	0.014	6	15	17	13	<0.001
	Active	17	10.8	68	60.0		62	51.7	23	19.2		2	7.3	75	64.7	
**Cervical Asymmetric**	Inactive	10	8.3	3	2.5	<0.001	9	7.5	39	32.5	<0.001	6	5	10	8.3	<0.001
	Active	18	20.0	89	69.2		18	13.3	54	46.7		35	29.2	69	57.5	
**Cervical Symmetric**	Inactive	24	20.0	12	10.0	<0.001	29	24.2	10	8.3	0.393	18	15	21	17.5	0.055
	Active	34	28.3	50	41.7		54	45.0	27	22.5		23	19.2	58	48.3	

N, Normal; B, blocked; AF, absolute frequency; RF, relative frequency (%).

## Data Availability

Not applicable.
